# Supplementary material for: Combination of Xpert® MTB/RIF and DetermineTM TB-LAM Ag improves the diagnosis of extrapulmonary tuberculosis at Jimma University Medical Center, Oromia, Ethiopia
Source: PLoS One. 2022 Feb 3;17(2):e0263172. doi: 10.1371/journal.pone.0263172 (PMC8812938; doi:10.1371/journal.pone.0263172)
Supplement: S1 File — (RTF) [file pone.0263172.s001.rtf]

Out of 23 HIV positive cases, 6 were EPTB positive by CRS. Smear microscopy and Xpert MTB/RIF detected only 2 and 3 of the 6 HIV and EPTB co-infected cases with a sensitivity of 33.3% and 50% respectively. However, TB-LAM detected 5 of the 6 HIV and EPTB co-infected cases with a sensitivity of 83.3%.  TB-LAM detected EPTB in additional 3 and 2 HIV infected patients who were missed by smear microscopy and Xpert MTB/RIF respectively.

Of the total 111 HIV negative presumptive EPTB cases, only 96 provided urine specimen for TB-LAM test.  By the CRS, 28 of the 111 HIV negative cases were diagnosed as EPTB. Out of 28 EPTB cases, 1 patient didn't provide urine specimen for TB-LAM test. In HIV negative cases, Xpert MTB/RIF detected 10 EPTB positive cases with a sensitivity of 35.7% (10/28) and TB-LAM test detected 7 EPTB positive cases with a sensitivity of 26% (7/27). The combination of TB-LAM and Xpert MTB/RIF test detected 14 EPTB positive cases of the total HIV negative cases with a sensitivity of 52% (14/27).        
