# Supplementary material for: Combination of Xpert® MTB/RIF and DetermineTM TB-LAM Ag improves the diagnosis of extrapulmonary tuberculosis at Jimma University Medical Center, Oromia, Ethiopia
Source: PLoS One. 2022 Feb 3;17(2):e0263172. doi: 10.1371/journal.pone.0263172 (PMC8812938; doi:10.1371/journal.pone.0263172)
Supplement: Attachment — Submitted filename: Response to Reviewers.docx [file pone.0263172.s003.docx]

**Response to Editor and Reviewers**

We appreciate the editor and reviewers for the constructive comments which we have used to improve the quality of the manuscript. We have re-written some portions of the manuscript accordingly. We have carefully addressed the comments line by line as follows.

**Response to Editor’s comments:**

**Comment 1**: Please ensure that your manuscript meets PLOS ONE's style requirements, including those for file naming

- **Response 1:** Thank you for your comment**.** We have accepted your comments and correction has made accordingly.

**Comment 2:** Please provide additional details regarding participant consent. In the ethics statement in the Methods and online submission information, please ensure that you have specified whether consent was informed.

- **Response 2:** Written informed consent was obtained from each participants before enrolling into our study. This was described method parts.

**Comment 3:** We note that you have indicated that data from this study are available upon request. PLOS only allows data to be available upon request if there are legal or ethical restrictions on sharing data publicly. If there are ethical or legal restrictions on sharing a de-identified data set, please explain them in detail and who has imposed them. Please also provide contact information for a data access committee, ethics committee, or other institutional body to which data requests may be sent. If there are no restrictions, please upload the minimal anonymized data set necessary to replicate your study findings as either Supporting Information files or to a stable, public repository and provide us with the relevant URLs, DOIs, or accession numbers.

- **Response 3:** Thank you for your feedback. There is no legal restriction to share our data set. We uploaded our data set as a supporting information file (**S2 File**).

**Comment 4:** PLOS requires an ORCID iD for the corresponding author in Editorial Manager on papers submitted after December 6th, 2016. Please ensure that you have an ORCID iD and that it is validated in Editorial Manager.

- **Response 4:** Thank you for your comment. This is the ORCID iD of the corresponding author <https://orcid.org/0000-0003-4751-2225>.

**Comment 5:** We note that you have included the phrase “data not shown” in your manuscript. Unfortunately, this does not meet our data sharing requirements. PLOS does not permit references to inaccessible data. We require that authors provide all relevant data within the paper, Supporting Information files, or in an acceptable, public repository. Please add a citation to support this phrase or upload the data that corresponds with these findings to a stable repository (such as Figshare or Dryad) and provide and URLs, DOIs, or accession numbers that may be used to access these data. Or, if the data are not a core part of the research being presented in your study, we ask that you remove the phrase that refers to these data.

- **Response 5:** We agree with reviewer’s comment and the phrase referring the missed data was deleted from the manuscript.

**Additional editor question 1:** Line 188- 'Forty-nine (33.3%) of the presumptive EPTB cases had the classification of colitis'-On what basis was this classification made and what was the sample collected from these patients?

- **Response1:** About 49(33.3%) of the presumptive EPTB cases were classified as colitis based on the site of infection and symptoms. The site of infection for these 49 presumptive EPTB cases were abdomen and almost all of them experienced abdominal discomfort and pain during specimen collection. The sample collected from them were peritoneal fluid. So, due to the site of infection, abdominal discomfort and pain symptoms we have classified them as colitis.

**Additional editor question 2:** Although 4 of the 23 HIV positive patients were bacteriologically confirmed, it is not clear how many were positive by Xpert MTB/RIF. What is meant by 50% in Line 211? Similarly, for Determine TB LAM assay.

- **Response 2:** Thank you for your comment and question. Although not described in the document, out of 23 HIV positive cases 3 of them were positive by Xpert MTB/RIF. Out of 23 HIV positive cases only 21 provided urine specimen and 7 were positive by TB LAM. Out of 23 HIV positive cases 6 were positive for EPTB by composite reference standard (CRS) and out of these 6 cases, only 3 were positive by Xpert MTB/RIF. So, the sensitivity of Xpert MTB/RIF for HIV positive cases were 3/6 (50%). The detection rate and diagnostic performance of Xpert MTB/RIF, TB-LAM and their combination in HIV-positive and –negative patients is now described in the supporting information file (S1 File).

**Additional editor question 3:** Line 320- How was the sensitivity of LJ culture derived to be 62.2%? What was the gold standard? I think that was not within the scope of the study.

- **Response 3:** Of course, determining the sensitivity of LJ culture is not our primary objective. Previous studies have shown that LJ culture has suboptimal sensitivity for diagnosis of EPTB and there are a significant number of cases which are negative on LJ culture but clinically diagnosed as TB. Taking note of this, we proposed to determine the sensitivity of LJ culture using CRS (composite reference standard) as a gold standard. In our study, a total of 37 patients were classified as EPTB based on CRS, of whom only 23 were positive by LJ culture. Thus, the sensitivity of LJ culture was 23/37(62.2%). We kept this just as an additional information.

**Additional editor comment 4:** Fig 1- The flow chart could start with inclusion of 147 patients suspected of presumptive TB instead of 160 patients.

- **Response 4:** Thank you for your comment. We have accepted your comment and it was corrected on the revised manuscript.

**Response to Reviewers' comments**

**Reviewers' comment 1:** In the current study the authors aim to investigate the diagnostic performance of combined use of Xpert MTB/RIF and TB LAM Ag test for detection of M. tuberculosis complex among EPTB patients in a medical center of Ethiopia. Considering the high prevalence of EPTB and association of HIV in Ethiopia, the current diagnostic approach provides the important insight for increased detection of EPTB cases in clinical settings. However, the current manuscript needs to address several issues before approval.

- **Response 1:** We thank the reviewer for the constructive feedback. We tried our best to address comments/questions raised by the editor and reviewers.

**Response to Reviewer #1 Major Comments**

**Comment 1:** According to the manufacturer (Abbot Laboratories, Chicago, US), the test name is ‘Determine™ TB-LAM Ag’. It is suggested to use the exact test name in the Title of the manuscript. Throughout the text, authors should be consistent mentioning the test name. Examples of some discrepancies are: ‘Determine TB LAM test’ in Line-31, ‘TB LAM’ in Line 41, ‘Determine TB-LAM test’ in line 136, ‘TB-LAM assay’ in line 140 and so on. It is suggested to use ‘Determine™ TB-LAM Ag (TB-LAM)’ when appears first in the text, followed by ‘TB-LAM’ all through the text.

- **Response 1:** Thank you for your comment. In the revised manuscript, this was corrected.

**Comment 2:** In the introduction section, please add one paragraph describing the other published data on the performances of the Xpert MTB/RIF assay and Determine TB-LAM assay for detection of EPTB cases.

- **Response 2**: We agree with reviewer’s comment and more evidence on the performance of Xpert MTB/RIF and TB-LAM was included in the introduction section of revised version.

**Comment 3:** One of the EPTB samples as has been described inconsistently throughout the text. For example, in Table-1 as ‘pus (abscess)’, Table-2 and throughout the text as ‘Abscess’. According to Line-191, it appears that samples were obtained from ‘Lymph node TB’. It would be easier for the readers to understand if the term ‘Lymph node aspirate’ is used all through the manuscript instead of ‘Abscess’.

- **Response 3:** Thank you for your comment. We agree with reviewer’s comment and it was corrected as lymph node aspirate in the revised manuscript.

**Comment 4:** Gastric aspirate is considered as an alternative specimen for detection of pulmonary TB when patients cannot expectorate the sputum. As this study aims to detect the EPTB cases, authors should exclude the two ‘gastric aspirate’ samples from the study and re-analyze the results and correct the statistics accordingly in different relevant Tables, Figures and throughout the text.

- **Response 4:** Thank you for the interesting feedback. As the reviewer mentioned, in most cases, gastric aspirate is an alternative specimen for detection of pulmonary TB when patients cannot expectorate the sputum. But, in our case, the 2 gastric aspirate specimens were taken from patients who were suspected of having disseminated tuberculosis that is gastro intestinal tuberculosis.

**Comment 5:** For Table-2 and Table-3: Authors should check carefully that the statistics are presented correctly. It seems that the value of sensitivity, specificity, PPV and NPV and their corresponding 95% CI are not accurate. As for example, in Table-2 (based on the calculation of Fig-1), for ‘Determine TB LAM test’: the sensitivity would be 34.8% with 95% CI ranging from 16.4-57.3, specificity would be 92.7% (86.7-96.6), PPV would be 47.1% (27.7-67.4), and NPV would be 88.5% (85.0- 91.2). Please check both table 2 and 3, and correct accordingly. Please also add the 95% CI for specificity of Xpert MTB/RIF test in both tables. Should use the value of 95% CI in a consistent format like 0.0% (0.0-0.0) instead of 0.0% (0.0%-0.0%) in the tables and text.

- **Response 5:** The reviewer’s comments are well taken and were corrected in the revised manuscript. The sensitivity, specificity, positive predictive value and negative predictive value for Xpert MTB/RIF and Smear microscopy were calculated from 147 participants but for TB-LAM the participants were 126.

**Comment 6:** For clarity and better understanding authors should include the number of samples detected or not detected by the index tests compared to the reference tests along with the value of sensitivity and specificity. As for example, in Table-2, for ‘Determine TB LAM test’ please write the Sensitivity: 34.8% (34.1-35.5); 8/23 instead of 34.8% (34.1-35.5), and for specificity: 92.7% (86.7-96.6); 115/124 instead of 92.7% (86.7-96.6). Please add the numbers in Table-2 and Table-3 and describe accordingly in the text.

- **Response 6:** We agree with what the reviewer suggested. In the revised version of the manuscript, the number of samples tested positive or negative by index test compared to the reference standard along with the values of sensitivity, specificity and predictive values are mentioned.

**Comment 7:** Figure 2: please describe in the result section (by mentioning the numbers of samples) how the percentages of different tests were obtained between HIV positive and negative patients

- **Response 7:** Details of the percentages obtained for Xpert MTB/RIF and TB-LAM tests between HIV-positive and –negative cases are well addressed in the revised version of the manuscript. The detection rate and diagnostic performance of TB-LAM, Xpert MTB/RIF and combination of TB-LAM and Xpert MTB/RIF between HIV-positive and -negative participants was also addressed in supporting file (**S1 File**).

**Comment 8**: Line 215-217: It is not clear how the sensitivities of 44.4% for Xpert and 30.6% for TB LAM were obtained. Please clarify. Please also mention the statistical methods in ‘Methods and Material’ section that was used to obtain the p value.

- **Response 8:** There were 126 patients who provided both site-specific extrapulmonary specimen (for Xpert MTB/RIF) and urine specimen (for TB-LAM). Comparison of Xpert MTB/RIF and TB-LAM was done only for 126 samples. Out of the 126 cases, about 36 were positive for EPTB by composite reference standard (CRS). Xpert MTB/RIF detected only 16 EPTB cases out of the 36 EPTB positive cases. So, the sensitivity of Xpert MTB/RIF test to detect EPTB among 126 cases were 16/36(44.4%). TB-LAM test detects only 12 EPTB cases out of the total EPTB positive cases (36). So, the sensitivity of TB-LAM test to detect EPTB among 126 cases were 12/36(33.3%). The sensitivity of TB-LAM test to detect EPTB among 126 cases was corrected on the revised manuscript from 30.6% to 33.3%.

**Comment 9**: Recently, Xpert MTB/RIF Ultra and Fujifilm SILVAMP TB-LAM have been appeared with higher sensitivities for detection of tuberculosis. In the discussion section authors should add one paragraph about the future applicability of these two assays compared with the current findings of Xpert MTB/RIF assay and Determine TB LAM assay.

- **Response 9:** We appreciate the reviewer for the insightful complement. Regarding the Xpert MTB/RFI Ultra, we have a bit discussed the potential added value of Ultra over the Xpert MTB/RIF particularly in paucibacillary samples in the second paragraph of discussion. As we have mentioned in the discussion, the better performance of Ultra over Xpert MTB/RIF is mainly due to its lower analytical limit of detection (LLD;15 CFU/mL for Ultra versus 100–120 CFU/mL for Xpert MTB/RIF). Somewhere in the discussion (at the end of 4^th^ paragraph), we also recommend the potential use of Xpert Ultra, when Xpert MTB/RIF yielded a negative result in body fluids such as pleural and peritoneal fluids. Regarding, the diagnostic yield of FujiLAM over TB-LAM, we also entertained this in the seventh paragraph of discussion as shown below’

***“****In their recent study, Kerkhoff et al. reported substantially higher sensitivity of Fujifilm SILVAMP TB-LAM (FujiLAM) over TB-LAM for detecting EPTB in HIV inpatients with moderate sensitivity in pleural fluid and CSF. This suggests a potential use of FujiLAM as a first-line test for the rapid detection of EPTB in HIV patients, with substantial added benefit in paucibacillary diseases such as pleural TB and TB meningitis.*’’

**Comment 10:** Line 344: Please clarify the statement ‘equivalent to culture to diagnose EPTB’. It is not clear how the sensitivity of combined use of Xpert MTB/RIF and TB LAM was equivalent to ‘culture’ as the authors did not mention anything about the sensitivity of ‘culture’ in the manuscript.

- **Response 10:** Thank you for your comment. In our study, LJ culture was positive for 23 cases out of the total 147 presumptive EPTB cases. Composite reference standard (CRS) detected 37 EPTB cases out of the total 147 presumptive EPTB cases. So, the sensitivity of L-J culture was 62.2% (23/37) that is equivalent to the previously calculated sensitivity of combined use of Xpert MTB/RIF and TB LAM test (61.1%). In the revised manuscript, we have mentioned the sensitivity of LJ culture.

**Comment 11:** While the text is readable, there are some grammar mistakes. Please correct the text to improve the comprehensibility.

- **Response 11:** Thank you for your comment. We have thoroughly gone through the manuscript and edited it for grammar mistakes.

**Response to Reviewer #1 Minor Comments**

**Line 37:**

**Comment 1:** The authors should mention the name of ‘reference’ test that is the ‘CRS’ to whom the results of Xpert MTB/RIF were compared.

- **Response 1:** We have accepted your comment and it was mentioned in the revised manuscript.

**Line 42:**

**Comment 2**: Please write ‘EPTB cases’ instead of ‘TB cases’

- **Response 2**: Comment accepted and corrected in the revised manuscript.

**Line 41-42:**

**Comment 3:** Please correct and re-phrase the sentence ‘The combination of Xpert MTB/RIF and TB LAM detected 61.1%of all EPTB participants and 83.3% of HIV co-infected TB cases’ by mentioning the sensitivity instead of detection.

- **Response 3:** The reviewers’ comments are noted and corrected in the revised manuscript.

**Line 98:**

**Comment 4**: Should write Lowenstein Jensen (L-J) as it appears first in the text.

- **Response 4:** Comment accepted and corrected on the revised manuscript.

**Line 135:**

**Comment 5:** Please mention the volume of urine used for centrifugation.
Authors should specify why the LAM test was performed on refrigerated urine samples instead of freshly collected samples. Should discuss the point in the discussion section whether the sensitivity of LAM test varies between refrigerated versus fresh urine sample.

## **Response 5:** As it has been mentioned in the **Materials and Methods** section, under the subheading of **study subjects, sample collection and laboratory tests**, all participants were requested to submit 10-30ml of urine sample, which was immediately refrigerated upon arrival to Mycobacteriology Research Center. Since there was a delay in the shipment process of TB-LAM kit, in our study TB-LAM test was done on the refrigerated urine samples. Though not reproduced by other researchers, Peter *et al.* has reported that the use of frozen urine has been associated with reduced TB- LAM sensitivity. We have mentioned the use of frozen urine sample as the possible limitation of our study and we have indicated that the low sensitivity of TB-LAM in our study could be partly because of the fact that the test was done on frozen urine sample.

**Line172:**

**Comment 6**: Please write as 35 years (IQR, 22-45).

- **Response 6:** Comment noted and corrected on the revised manuscript.

**Line-187:**

**Comment 7:** ‘Forty-nine (33.3%) of the presumptive EPTB cases had the classification of colitis’. Please clarify whether all of the 49 cases from where peritoneal fluid were collected had ‘colitis’

- **Response 7:** Thank you for your comment. Out of 147, about 49(33.3%) of the presumptive EPTB cases were classified as colitis based on the site of infection and symptoms. Peritoneal fluid was collected from all (49) cases and all of them experienced abdominal discomfort and pain during specimen collection so that all of them were diagnosed as colitis.

**Line-195 and 202:**

**Comment 8:** Authors should mention ‘detection rate’ instead of ‘prevalence’. Authors have mentioned in Table-1 that one sample among ‘Others’ group (2 gastric aspirates and 2 synovial fluids) was diagnosed as ‘Confirmed TB’. Please, specifically mention the sample type (either the gastric aspirate or synovial fluid) that was positive for TB in the table and add the description in line-196.

- **Response 8:** We also agree with reviewers’ comments and considered in the revised version of the manuscript.

**Line 228-229:**

**Comment 9:** please write as (0.0%; 95% CI, 0.0-0.0) instead of ‘(30.6% (95%CI, 30%- 229 31%))’ and ‘(93.3% (95%CI, 93.2%-93.5%))’.

- **Response 9:** We agree with reviewer’s suggestion and corrected accordingly.

**Line 230 and 238:**

**Comment 10:** Authors should include the ‘p value’ of the significant difference, and the test used.

- **Response 10:** Comment well accepted and corrected in the revised manuscript.

**Line 235-236:**

**Comment 11**: Please rephrase the sentence ‘Adding Determine TB LAM test to Xpert MTB/RIF test increases the sensitivity of Xpert MTB/RIF test from 43.2% to 61.1%’ as it does not increase the sensitivity of Xpert MTB/RIF test, but increases the sensitivity of combined use TB LAM and Xpert assay.

- **Response 11:** Thank you for the interesting comment. We have now rephrased the sentence as “The combined use of TB- LAM and Xpert MTB/RIF tests had the sensitivity of 61.1% when compared to CRS, which was significantly higher than the sensitivity of each test alone”

**Line 307-308:**

**Comment 12:** It is not clear how the values of 2 (33%) and 1 (17%) were obtained. Please add in the result section to clearly state how these values were obtained.

- **Response 12:** We also agree with reviewer’s feedback. We found that it is better to describe the diagnostic performance of Xpert MTB/RIF, TB-LAM and their combined use among HIV-positive and negatives case, and we added one subheading in the result section. By including this, we assumed we have addressed some of questions/concerns raised from the reviewer.

**Line 320:**

**Comment 13:** It is not clear how the sensitivity of L-J culture (62.2%) was obtained. Please specify.

- **Response 13:** We have already addressed this question above in **Response 10 under Major comments**. To clarify it once again, of the total 37 EPTB cases diagnosed by CRS (the reference standard method used in our study), 23 were identified as EPTB positive by L-J culture. Using CRS (definite and probable-EPTB cases) as a gold standard, the sensitivity of L-J culture was 62.2% (23/37).
