# Supplementary material for: Combination of Xpert® MTB/RIF and DetermineTM TB-LAM Ag improves the diagnosis of extrapulmonary tuberculosis at Jimma University Medical Center, Oromia, Ethiopia
Source: PLoS One. 2022 Feb 3;17(2):e0263172. doi: 10.1371/journal.pone.0263172 (PMC8812938; doi:10.1371/journal.pone.0263172)
Supplement: Attachment — Submitted filename: Response to Reviewers.docx [file pone.0263172.s004.docx]

**Response to Reviewers**

We appreciate the reviewers for the constructive comments which we have used to improve the quality of the manuscript. As we did in the last time, we have re-written some portions of the manuscript accordingly. We have also carefully addressed the reviewers’ comments line by line as follows:

**Response to Reviewers' comments**

**Reviewer 1**

**Comment 1:** It is appreciated that the authors have responded well the comments. However, it would be easier for the reviewer to go through the text if the specific line numbers are mentioned against the comments (where appropriate) in the cleaned version.

- **Response 1:** Sorry for not mentioning the specific line numbers against the comments in the last cleaned version. We agree with the reviewer’s comment. Now, in the recent version, the specific line numbers are indicated against the comments in the cleaned version.

**Comment 2:** From **Table 2,** it is evident that TB-LAM test was compared against 36 and Xpert MTB/RIF test against 37 positive samples found by CRS. Would be easier for the reader if it is explained in short in the result section how these CRS have been found.

- **Response 2:** In general, as indicated in the method section (**Line 166-169** ), CRS, which comprises of smear microscopy, L-J culture and clinical improvement after ATT initiation, was used as gold standard to calculate the sensitivity, specificity and predictive values of both TB-LAM and Xpert MTB/RIF. Specifically for calculation of sensitivity, as it was stated by the reviewer, TB-LAM test was compared against 36 CRS-positive cases whereas Xpert MTB/RIF was compared against 37 CRS-positive cases. This is now addressed in the revised version of the manuscript. **See Line224-226 of cleaned version of the manuscript.**

**Comment 3:** Line 227: the author should write ‘sensitivity’ instead of ‘pooled sensitivity’

- **Response 3:** We have modified it accordingly. **Shown in Line 232.**

**Comment 4:** Line 179-180: ‘MedCalc Software Ltd (Comparison of proportions calculator) was used……….’ this sentence can be written as ‘Comparison of proportion between the methods was done by Chi square test using the ‘MedCalc Software’ (please include the weblink).

- **Response 4:** We agree with the reviewer’s feedback and we have modified it accordingly as shown **Line 177-180 of cleaned version of the manuscript**	.

**Reviewer 2**

**Comment 1:** The referencing style need to be uniform throughout the manuscript in accordance with Journal’s requirement. For example, please note the discrepancy in citing reference no. 1, 7, 13, 20, 24

- **Response 1:** The reviewer’s comment is accepted and corrected accordingly**.**

**Comment 2: In ABSTRACT;**

- **Comment 2.1: Line 29** of the revised manuscript: Replace “examined for tuberculosis (TB)” with “tested for *Mycobacterium tuberculosis* complex (MTBC)”.
  - **Response 2.1:** Comment accepted and corrected accordingly. **Shown in Line 27 of cleaned version.**
- **Comment 2.2: Line 40** of the revised manuscript: Write “and 100% specificity” instead of “with 100% specificity”.
  - **Response 2.2:** Comment accepted and corrected accordingly. **Shown in Line 38 of cleaned version.**

**Comment 3: In BACKGROUND**

- **Comment 3.1: Line 60** of the revised manuscript: Replace “category III biosafety level” with “bio-safety level III facilities”.
  - **Response 3:** We agree with the reviewer’s suggestion and corrected accordingly. **Shown in Line 58 of cleaned version.**

**Comment 4: In MATERIALS AND METHODS**

- **Comment 4.1**: Line 106-107 of the revised manuscript: There is no mention about collection of ‘gastric aspirate’ and ‘synovial fluid’.
  - **Response 4.1:** Gastric aspirate and synovial fluid samples were collected as per the standard of care. Information on the collection of synovial fluid and gastric aspirate is shown in **Line 104-105 of cleaned version.**
- **Comment 4**.2: Line 121-122 of the revised manuscript: Was decontamination also carried out for gastric aspirate specimens?
  - **Response 4.2:** Of course, like that of lymph node aspirates and blood stained samples, gastric aspirate samples were decontaminated by the standard *N*-acetyl-L-cysteine and sodium hydroxide (NALC/NaOH) to reduce overgrowth from contaminating microorganism. This was addressed in the revised cleaned version. **See Line 119 of cleaned version**
- **Comment 4**.**3**: Line 179-180 of the revised manuscript: Earlier minor comment no. 10 by the reviewer is not addressed adequately. The tool used in determining the statistical difference has been mentioned but the statistical test used for the same has not been mentioned in the revised version of the manuscript.
  - **Response 4.3:** As we have explained in the revised version (**Line 177-180),** presence or absence of statistically significant difference is determined based on the *P*-value. The *P*-value<0.05 showed statistically significant difference whereas the *P*-value>0.05 showed no difference (similar, not merely the same).

**Comment 5: In RESULTS**

- **Comment 5.1:** Line 206-107 of the revised manuscript: With respect to addition editor’s question no. 1 and the reply by the authors, will it be better if the authors mention the ‘colitis’ cases as ‘suspected abdominal TB’ cases because abdominal pain and discomfort could be due to causes other than colitis?
  - **Response 5.1:** We agree with the feedback from the reviewer and corrected it. **See Line 206-207 of cleaned version.**
- **Comment 5.2:** Line 215-216 of the revised manuscript: “1 disseminated TB from gastric aspirate”- It remains dubious how detecting MTBC from gastric aspirate only can label the case as ‘disseminated TB’. Clarification is needed.
  - **Response 5.2:** This is an interesting observation from the reviewer. We strongly agree with the reviewer’s concern and one MTBC case from gastric aspirate cannot signify disseminated TB. However, pulmonary manifestation was excluded from this patient and we assumed this patient had extrpulmonary form of TB. Accordingly, we revised the manuscript as shown in **Line 215-217 of cleaned version.**
- **Comment 5.3:** Line 242-243 of the revised manuscript: It is not clear whether culture confirmation (23 patients) could only be done only among 126 presumptive EPTB cases who submitted urine samples for TB-LAM. In other words, was there no culture confirmed cases among the other 21 presumptive EPTB cases who did not submit urine sample for TB-LAM? This needs to be clarified.
  - **Response 5.3:** This is again another interesting comment from the reviewer. The other reviewer also raised similar concern**.** There was one culture-positive case from presumptive cases who did not submit urine sample. That could be why we excluded this case from CRS when determining the diagnostic accuracy of TB-LAM. As it was shown in **Table 2**, the TB-LAM test was compared against 36 CRS positive cases whereas Xpert MTB/RIF test was compared against 37 CRS positive cases. For combination of TB-LAM and Xpert MTB/RIF, the 36 cases who have all the three test results were considered. **Shown in Line 224-226 of cleaned version.**

**Comment 6: In DISCUSSION**

- **Comment 6.1:** Line 316 of the revised manuscript: Replace ‘Additionally’ with ‘However’.
  - **Response 6.1:** We agree. This is corrected in the revised version of the manuscript as shown in **Line 315.**
- **Comment 6.2:** Line 355 of the revised manuscript: Write ‘End TB strategy’ instead of ‘end TB strategy’.
  - **Response 6.2:** It is written as End TB strategy (**Line 358**) in the revised version of the manuscript.

**Comment 7:** PLOS authors have the option to publish the peer review history of their article (what does this mean?). If published, this will include your full peer review and any attached files.

- **Response 7:** We are OK with the option to publish the peer review history.
